# Regional variation in use of exogenous and endogenous glomerular filtration rate (GFR) markers in Sweden

**DOI:** 10.3109/03009734.2012.664179

**Published:** 2012-08

**Authors:** Susanne Vilhelmsdotter Allander, Lars-Åke Marké, Björn Wihlen, Maria Svensson, Carl-Gustaf Elinder, Anders Larsson

**Affiliations:** ^1^SBU, Stockholm, Sweden; ^2^Department of Molecular and Clinical Medicine, Sahlgrenska University Hospital, Gothenburg, Sweden; ^3^Nephrology Unit, Department of Clinical Sciences Intervention and Technology, Karolinska Institute, Stockholm, Sweden; ^4^Department of Medical Sciences, Uppsala University, Uppsala, Sweden

**Keywords:** Clinical chemistry tests, diagnostic tests, family practice, glomerular filtration rate, health care costs, physician's practice patterns

## Abstract

**Background:**

Markers of renal function (glomerular filtration rate (GFR)) are frequently used in the Swedish health care. GFR is usually estimated based on plasma creatinine concentration, but plasma cystatin C concentration, creatinine clearance, iohexol clearance, and ^51^Cr-EDTA clearance are also used. These markers are all part of the daily patient care, but there is little specific information on the clinical use of these markers. The aim of this study was to compare the use of these various GFR markers in different parts of Sweden and potential changes over time.

**Methods:**

Retrospective study using questionnaires to collect information for the years 2006–2009 divided per county on the specific use of GFR markers with type of test reports.

**Results:**

Plasma/serum creatinine concentration (96%) is by far the dominating GFR marker in Sweden, while cystatin C concentration (3.5%), creatinine clearance (0.1%), iohexol clearance (0.1%), and 51Cr-EDTA clearance (0.1%) are less frequently used. The use of GFR markers, including creatinine, continues to increase on a national level with the exception of creatinine clearance and 51Cr-EDTA clearance. There were considerable variations between different counties in the use of GFR markers and the type of test reports that the laboratories provided.

**Conclusions:**

The inter-county variations of GFR markers used in Sweden are large and indicate that savings associated with optimized test utilization in this regard could be substantial. Regional habits and traditions are likely to influence the variations in GFR marker use.

## Introduction

Glomerular filtration rate (GFR) describes the flow rate of filtered blood through the kidney; it is generally accepted as the best overall indicator of renal function and is therefore an important marker for renal disease (1–4). Determination of GFR is an essential part of modern health care, and it is used in all medical areas.

Precise determination of GFR requires the measurement of exogenous substance that is freely filtered by the kidney and does not undergo metabolism, tubular secretion, or absorption (5). Inulin (urinary clearance) appears to be the molecule that best fulfils these criteria when given as a continuous infusion (1). However, inulin clearance is not practical for clinical routine purposes. Inulin has thus been replaced in routine health care by other exogenous markers such as the radiolabelled markers ^99m^Tc-diethylenetriamine penta-acetic acid (DTPA), ^169^Yb-DTPA, ^125^I-iothalamate, and ^51^Cr-ethylenediamine tetra-acetic acid (EDTA) or the non-radioactive contrast medium iohexol. ^51^Cr-EDTA and iohexol are the leading exogenous markers of GFR in Sweden. In most cases even these markers are considered impractical and too costly, and endogenous GFR markers are thus used instead. The first developed and today the most widely used endogenous GFR marker is serum creatinine concentration (1). Alternatives to serum creatinine concentration are endogenous urinary creatinine clearance and serum cystatin C concentration. Both creatinine and cystatin C can be performed on routine chemistry analysers providing 24-h availability with short test turnaround times. Creatinine clearance requires collection of urine, preferably for 24 h, which prohibits rapid test results. Creatinine, cystatin C, creatinine clearance, iohexol clearance, and Cr-EDTA clearance are all performed at Swedish laboratories, but we have limited knowledge on the actual use and if there are regional differences in use. Also, GFR measurements can either be reported as mL/min (absolute GFR) or as mL/min/1.73 m^2^ (relative GFR) (1). Test results in mL/min are mainly used for drug dosage while test results in mL/min/1.73 m^2^ are more focused towards kidney diseases. Traditionally the laboratories report serum creatinine only as a concentration (µmol/L), and the physician receiving the test results is thus expected to estimate GFR from the creatinine concentration using Cockcroft–Gault, Modification of Diet in Renal Disease Study (MDRD), or Chronic Kidney Disease Epidemiology Collaboration (CKD-EPI) equations (6,7). These equations are complex and thus require a calculator or computer to provide reliable results. There are also web pages that can be used to calculate eGFR from a creatinine concentration (e.g. http://mdrd.com/or http://www.egfr.se/eGFRen.htm). To avoid erroneous eGFR results due to manual estimates it has been recommended that laboratories automatically should report eGFR values together with concentrations of creatinine or cystatin C (4). It is not known to what extent Swedish laboratories automatically report eGFR and which equations they use.

GFR estimates are routinely used in health care, and the increasing numbers of tests are associated with rapidly growing costs (8). Other studies have shown that many of the tests ordered are superfluous, and excess test ordering represents as much as 25%–40% of all tests (9), and 20%–95% of selected tests (10). Excessive testing leads not only to increased direct and indirect costs, but also causes unnecessary patient discomfort (11) and increases the risk of generating false positive test results (12), which may in turn cause unnecessary worry, further investigations, and may thus be harmful to patients (13). Optimized use of laboratory analyses is one way of controlling the costs while maintaining the quality of the care provided (14).

The aim of the present study was to evaluate the utilization of endogenous and exogenous GFR markers in Swedish counties. Large variation of test utilization indicates that it should be possible to improve and optimize the use of GFR tests.

## Materials and methods

A questionnaire containing 20 questions on GFR markers were sent to clinical chemistry laboratories at Swedish hospitals. Questions included the number of creatinine, cystatin C, endogenous creatinine clearance, and iohexol clearance tests performed during 2006–2009. Information on 51Cr-EDTA clearance was obtained from *Strålskyddsmyndigheten* (a national authority). Two of the counties could not provide production statistics for 2006. Therefore, the trend analysis of GFR use was limited to years 2007–2009.

The aim of the questionnaire was to gain information on the use of GFR markers in different parts of Sweden. Results are presented by county. Point-of-care tests (POCT) for GFR that were not registered in the laboratory information systems were excluded from the study. Of the GFR markers, only creatinine is available as a POCT in Sweden, and the number of POCT creatinine assays is low in relation to the total number of creatinine tests. At some hospitals POCT for creatinine are included in the blood gas panels at intensive care units. As they are given together with blood gases, it is also difficult to know if creatinine results were actually used for patient care. It is thus difficult to get an accurate estimate for these POCT assays, and they were excluded from the comparison. Cost estimates were expressed in euros at an exchange rate of €1 = SEK 10.

**Table I.** Total number of plasma/serum creatinine and cystatin C analyses, endogenous creatinine clearances, exogenous iohexol, and 51Cr-EDTA clearances performed in Sweden during 2007–2009.

Type of analysis	2007	2008	2009
Creatinine	5,159,208	5,462,833	5,620,087
Cystatin C	114,144	165,672	196,331
Creatinine clearance (with urine collection)	17,127	15,127	12,077
Iohexol clearance	8,964	9,383	9,976
51Cr-EDTA clearance	6,232	5,509	5,345

## Results

### Use of endogenous and exogenous GFR markers in 2009

**Figure 1.**
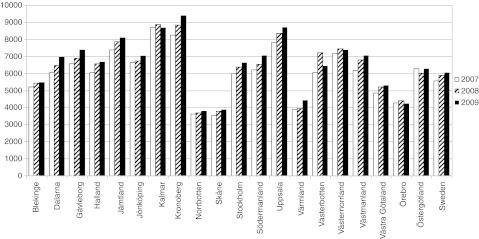
Number of creatinine assays per 10,000 inhabitants for 2007–2009. The figures are presented per county.

In 2009 there were a total of 5.6 million (96%) plasma/serum creatinine assays and 196,000 (3.5%) cystatin C assays performed in Sweden. The number of iohexol clearance tests was 7,204 (0.1%), while the number of 51Cr-EDTA clearance was 5,345 (0.1%), and the number of creatinine clearance was 6,577 (0.1%) (Figure 1 and Table I).

All counties but five used enzymatic creatinine assays. These five laboratories were using Jaffe-based creatinine methods. All but three counties were using Equalis nation-wide standardization of their creatinine methods.

Only six counties automatically reported creatinine results as estimated GFR. The laboratories that reported creatinine-based eGFR used the Lund–Malmö equation (Skåne), the isotope dilution mass spectrometry (IDMS)-calibrated MDRD equation (three counties), or the original MDRD equation (one county).

One county also reported eGFR using the Cockcroft–Gault equation. All counties but one reported cystatin C as eGFR. All counties but one reported relative GFR as mL/min/1.73 m^2^.

### Changes in GFR between the years 2007 and 2009

Creatinine showed the largest increase in absolute numbers, while cystatin C showed the proportionally largest increase (%) (Table I). Creatinine analyses increased by approximately 5% per year despite creatinine already being a well-established test. Endogenous urinary creatinine clearance and ^51^Cr-EDTA test numbers both decreased during the study period.

### Difference in GFR marker use between counties (Table II)

**Table II.** Differences between Swedish counties in the number of GFR markers performed during 2009. The figures are presented per 10,000 inhabitants.

	Creatinine	Cystatin C	Creatinine clearance	Iohexol-clearance	51Cr-EDTA-clearance
Blekinge	5470	25.1	0.9	0	0
Dalarna	6920	327	11.3	5.8	0
Gävleborg	7400	165	17	0	0.3
Halland	6750	74.8	0	18.7	0.3
Jämtland	8300	260	2.8	0	5.5
Jönköping	6910	344	0.7	18.2	0.1
Kalmar	8730	59.8	37.8	7.4	2.6
Kronoberg	9220	25.6	1	23.3	0
Norrbotten	3840	71	0	0	16.3
Skåne	3900	293	0	16	0.3
Stockholm	6390	183	26.5	9.2	0.2
Södermanland	6810	39.7	7.2	10.3	0
Uppsala	7880	1970	32.2	3.8	3
Värmland	4440	95.4	0	20.1	1.9
Västerbotten	6460	27.1	61.4	3.7	23.1
Västernorrland	7280	218	0.7	21.9	2.9
Västmanland	6970	44.6	19.9	8	0
Västra Götaland	5420	21.4	0	10	24.7
Örebro	4420	92.3	0.2	15.3	0
Östergötland	6270	129	33.8	9.4	0.1

**Figure 2.**
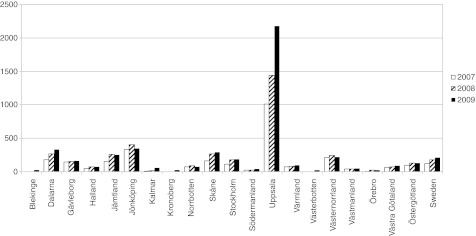
Number of cystatin C assays per 10,000 inhabitants for 2007–2009. The figures are presented per county.

The use of creatinine differed by more than a factor two between counties in Sweden. Kronoberg county (southern Sweden) performed almost 9400 creatinine assays/10,000 inhabitants while Skåne county (southern Sweden) had approximately 3900 creatinine requests/10,000 inhabitants (Figure 1). Uppsala county (Stockholm area) performed by far the most cystatin C tests (2180/10,000 inhabitants)(15,16) while some counties hardly performed any cystatin C assays at all (Figure 2).

Data for iohexol clearance and ^51^Cr-EDTA clearance seem to indicate that the counties prefer use of one or the other of these exogenous GFR markers. Västerbotten (northern Sweden) was the county that performed the largest number of urinary creatinine clearances (61/10,000 inhabitants).

### Total cost for exogenous and endogenous GFR markers in Sweden for 2009

The total cost for all counties was €14.3 million (excluding cost for blood sampling). Creatinine costs represented approximately 70% of the total cost.

The mean cost per inhabitant was €1.5 for Sweden, but there were significant variations between counties. The lowest cost for GFR markers was in Örebro (€0.86), Skåne (€0.89), and Värmland (€0.98), and the highest costs were found in Västra Götaland (€2.38), Västerbotten (€2.48), and Uppsala (€2.63).

## Discussion

Our study revealed an extensive use of GFR markers in Sweden corresponding to approximately 0.6 tests per year per inhabitant. Creatinine is by far the dominating GFR marker. It is surprising that such a ‘mature’ test continues to increase by 5% per year. There are no new indications that could explain this effect, but an increased awareness of chronic kidney disease and drug-related morbidity might have contributed to the increase. Another contributing factor could be the shift from the Jaffe (picric acid)-based method to a more precise and specific enzymatic method employed by a majority of the counties. However, there was no clear-cut indication that those counties that retained the Jaffe method were those who utilized the creatinine assays least. There were also large inter-county variations in creatinine use that are difficult to explain based on the differences in populations in the different counties.

Few of the Swedish laboratories provided automatic reporting of estimated GFR (eGFR) when analysing creatinine, despite the international recommendations to automatically report creatinine as eGFR (17). This may be due to the fact that a limited number of large studies verify the appropriateness of the US-based equations in a Swedish population. A Swedish study indicates differences between MDRD and iohexol clearance in Swedish patients (18). Skåne county (southern Sweden) has chosen a locally derived equation (the Lund–Malmö equation) that is well adapted for their population (19). Some of the other laboratories use the old MDRD equation (20) that with present Swedish creatinine methods will lead to an overestimation of GFR by 10%–30% (21).

There was a rapid increase in the use of cystatin C, and the regional differences for cystatin C were even more striking than those for creatinine. The regional variations can at least partially be explained by the fact that the assay is a new routine test. Factors that may influence the use of a new test are for instance price, availability, local information about the assay, and how the test results are presented. Uppsala county with the highest use of cystatin C has the same price and test turnaround time as for creatinine. The laboratory also introduced early on an automatic report system of cystatin C as eGFR.

In contrast to plasma/serum creatinine and cystatin C analyses, exogenous ^51^Cr-EDTA and endogenous creatinine clearances decreased during the observation period. The impression is that ^51^Cr-EDTA is at least partially replaced by iohexol as a marker. Both ^51^Cr-EDTA and iohexol are considered as good exogenous markers when measuring GFR. The advantage with iohexol is that the molecule is non-radioactive, but iohexol also has disadvantages as it may cause kidney injury at high concentrations. The doses used for iohexol clearance are much lower than those used in a radiological contrast medium context. Thus, the risk for kidney injury during iohexol clearance measurement is limited. The reduction in the number of endogenous creatinine clearance measurements is judged as favourable, as the method has a rather poor correlation with inulin clearance (22,23). Moreover, it is comparatively expensive if the total cost for 24-h urine collection is included. It is also impractical especially for out-patients that have to collect urine for up to 24 h.

The study revealed large regional differences in GFR test utilization in Swedish health care. Several non-evidence-based medical factors are held as explanatory to variations in test ordering (24). Differences in population between counties can not explain the regional differences in GFR marker use, and it is likely that both use and preference of GFR marker is dependent on local traditions. From this type of study it is not possible to state the optimal use, but large differences between counties indicate a clear potential for a cost/benefit analysis to improve especially the utilization of serum/plasma creatinine as a GFR marker within a county.
